# A Palmitic Acid-Conjugated, Peptide-Based pan-CoV Fusion Inhibitor Potently Inhibits Infection of SARS-CoV-2 Omicron and Other Variants of Concern

**DOI:** 10.3390/v14030549

**Published:** 2022-03-06

**Authors:** Qiaoshuai Lan, Jasper Fuk-Woo Chan, Wei Xu, Lijue Wang, Fanke Jiao, Guangxu Zhang, Jing Pu, Jie Zhou, Shuai Xia, Lu Lu, Kwok-Yung Yuen, Shibo Jiang, Qian Wang

**Affiliations:** 1 Key Laboratory of Medical Molecular Virology (MOE/NHC/CAMS), School of Basic Medical Sciences, Shanghai Frontiers Science Center of Pathogenic Microbes and Infection, Shanghai Institute of Infectious Disease and Biosecurity, Fudan University, Shanghai 200032, China; 18111010010@fudan.edu.cn (Q.L.); xuwei11@fudan.edu.cn (W.X.); 19111010061@fudan.edu.cn (L.W.); 20111010053@fudan.edu.cn (F.J.); 21111010088@m.fudan.edu.cn (G.Z.); 17111010015@fudan.edu.cn (J.P.); 19211010046@fudan.edu.cn (J.Z.); sxia15@fudan.edu.cn (S.X.); lul@fudan.edu.cn (L.L.); 2 Carol Yu Centre for Infection, State Key Laboratory of Emerging Infectious Diseases, Department of Microbiology, Li Ka Shing Faculty of Medicine, The University of Hong Kong, Pokfulam, Hong Kong, China; jfwchan@hku.hk; 3 Department of Clinical Microbiology and Infection Control, The University of Hong Kong-Shenzhen Hospital, Shenzhen 518000, China; 4 Centre for Virology, Vaccinology and Therapeutics, Hong Kong Science and Technology Park, Hong Kong, China

**Keywords:** palmitic acid, lipopeptide, Omicron, β-coronavirus, entry inhibitor

## Abstract

Our previous studies have shown that cholesterol-conjugated, peptide-based pan-coronavirus (CoV) fusion inhibitors can potently inhibit human CoV infection. However, only palmitic acid (C16)-based lipopeptide drugs have been tested clinically, suggesting that the development of C16-based lipopeptide drugs is feasible. Here, we designed and synthesized a C16-modified pan-CoV fusion inhibitor, EK1-C16, and found that it potently inhibited infection by SARS-CoV-2 and its variants of concern (VOCs), including Omicron, and other human CoVs and bat SARS-related CoVs (SARSr-CoVs). These results suggest that EK1-C16 could be further developed for clinical use to prevent and treat infection by the currently circulating MERS-CoV, SARS-CoV-2 and its VOCs, as well as any future emerging or re-emerging coronaviruses.

## 1. Introduction

Coronaviruses (CoVs) comprise a group of RNA viruses that can cause human or animal infection. Seven coronaviruses can infect humans, thus being named human CoVs (HCoVs), including severe acute respiratory syndrome coronavirus (SARS-CoV), severe acute respiratory syndrome coronavirus 2 (SARS-CoV-2), Middle East respiratory syndrome coronavirus (MERS-CoV), HCoV-229E, HCoV-OC43, HCoV-NL63, and HCoV-HKU1 [1]. Five belong to the β-CoV genus (i.e., sarbecoviruses), including SARS-CoV, SARS-CoV-2, MERS-CoV, HCoV-OC43, and HCoV-HKU1. Some SARSr-CoVs from bat (e.g., WIV1, Rs3367, and RsSHC014) also belong to the β-CoV genus.

SARS-CoV, SARS-CoV-2, and MERS-CoV, which belong to the group of highly pathogenic CoVs, possess high infectivity and transmissibility and can cause serious disease after infecting humans [2,3]. Since the end of 2019, when coronavirus disease 2019 (COVID-19) caused by SARS-CoV-2 was first reported, SARS-CoV-2 and its variants have infected about 3.6 billion individuals and caused more than 5.6 million deaths worldwide (https://covid19.who.int (accessed on 10 January 2022)). SARS-CoV-2 variants of concern (VOC), including Alpha, Beta, Gamma, Delta, and Omicron, have seriously compromised the clinical efficacy of many vaccines and antibody therapies [4,5,6], making it more difficult to control the COVID-19 pandemic.

In addition, low-pathogenic HCoVs in the β-CoV genus (HCoV-OC43 and HCoV-HKU1) usually cause the common cold in humans (mild upper respiratory tract infections) [7], but sometimes pneumonia in children, the elderly, or immunocompromised adults [8], calling for the development of more effective and broad-spectrum antivirals against both high- and low-pathogenic HCoVs [9,10,11].

An HCoV infects the host target cell through either a cytoplasmic or endosomal membrane fusion pathway. Each of these fusion processes occurs after the interaction of the receptor-binding domain (RBD) in the viral spike protein and cellular receptor, and proteolysis of spike protein mediated by transmembrane protease serine 2 (TMPRSS-2) on the cell surface or cathepsin L in the endosome. RBD-receptor interaction can be by RBD-specific antibodies and some mini-protein inhibitors [12], while the proteolytic function of TMPRSS-2 or cathepsin L can be inhibited by TMPRSS-2 inhibitors (e.g., camostat and nafamostat) or cathepsin L inhibitors (e.g., K11777), respectively [13].

**Figure 1:**
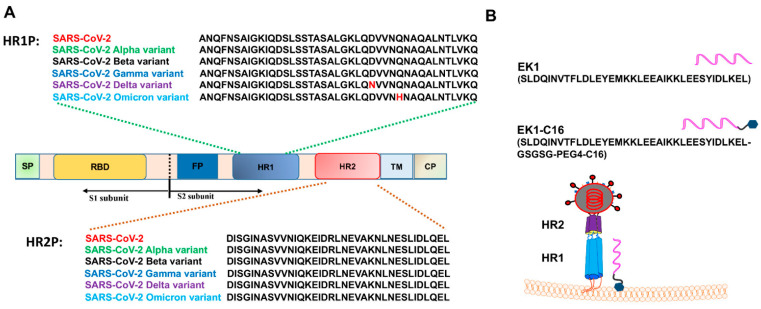
The amino acid sequences of HR1 and HR2 core regions of SARS-CoV-2 and its VOC, and design of EK1-C16 lipopeptide. (**A**) The amino acid sequences of HR1 and HR2 core regions of SARS-CoV-2 and its VOCs, including Alpha, Beta, Gamma, Delta, and Omicron variants. (**B**) Design of EK1-C16 lipopeptide and putative mechanism of potent antiviral activity of EK1-C16 lipopeptide. The C16 group of EK1-C16 can bind tightly with the cellular membrane of target cells, promoting the membrane-bound EK1-C16 peptide entering the endosome to inhibit the viral entry into the cytoplasm for replication, while lipid-free peptides only inhibit cytoplasm membrane fusion [24,25].

The six-helix bundle (6-HB) fusion core structure formed by HR1 and HR2 domains of SARS-CoV-2 is key for mediating membrane fusion. Previous studies confirmed its stability (Figure 1A), suggesting that the 6-HB fusion core is an important target for the development of pan-CoV fusion inhibitors against SARS-CoV-2 and its variants [14,15]. We and others demonstrated that peptides derived from the HR2 domain of SARS-CoV-2, such as 2019-nCoV-HR2P, IPB01, and SARS-CoV-2-HRC, could potently inhibit SARS-CoV-2 infection by interacting with the HR1 domain of SARS-CoV-2 S protein to block the formation of 6-HB fusion core between viral HR1 and HR2 domains [14,15,16]. In particular, our previously developed pan-CoV fusion inhibitor EK1 is effective against infection by SARS-CoV-2 D614G and its VOCs [17,18,19]. Later, we found that cholesterol- and 25-hydroxycholesterol-conjugated EK1 peptides, such as EK1C4 [17], EKL1C [20], and EK1P4HC [21], exhibited much improved antiviral activity against SARS-CoV-2, its VOCs, and other HCoVs, including SARS-CoV, MERS-CoV, HCoV-229E, HCoV-NL63, and HCoV-OC43, as well as bat SARSr-CoV WIV1, SARSr-CoV Rs3367, and SARSr-CoV SHC014. However, we note that no cholesterol-conjugated peptide drug is currently in clinical use, indicating the difficulty of developing clinically applicable cholesterol-based lipopeptide drugs. Interestingly, however, several palmitic acid-based lipopeptide drugs have been studied in clinical trials [22,23], suggesting the feasibility of their development.

Therefore, in this study, we designed and synthesized a palmitic acid (C16)-modified EK1 lipopeptide by adding a C16 group at the C-terminus of EK1 peptide, termed EK1-C16 (Figure 1B). We found that EK1-C16 could potently inhibit infection by SARS-CoV-2 wild-type strain (D614G) and its VOCs, including Alpha, Beta, Gamma, Delta, and Omicron, as well as other β-CoVs, including SARS-CoV, MERS-CoV, HCoV-OC43, and bat SARSr-CoV WIV1 and SARSr-CoV Rs3367. These results suggest that EK1-C16 is a potent lipopeptide-based pan-CoV fusion inhibitor with promise as an antiviral candidate with efficacy in preventing and treating infection by current circulating MERS-CoV and SARS-CoV-2 and its variants, as well as any future emerging or re-emerging coronaviruses.

## 2. Materials and Methods

### 2.1. Cell Lines, Plasmids, Peptides, and Viruses

HEK293T and Vero-E6 cell lines were obtained from the American Type Culture Collection (ATCC). Caco2, RD, and Huh-7 cell lines were obtained from the cell bank of the Chinese Academy of Science. All cell lines were cultured in Dulbecco’s Modified Eagle’s Medium (DMEM) containing 10% fetal bovine serum (FBS).

HIV-backbone plasmid (pNL4-3.Luc.R-E) and other plasmids coding the spike protein of coronaviruses (pAAV-IRES-GFP-SARS-CoV-2-D614G-spike, pAAV-IRES-GFP-MERS-CoV-spike, pcDNA3.1-SARS-CoV-2-spike, pcDNA3.1-SARS-CoV-2-B.1.1.7-spike, pcDNA3.1-SARS-CoV-2-P.1-spike, pcDNA3.1-SARS-CoV-2-B1.351-spike, pcDNA3.1-SARS-CoV-2-B1.617.2-spike, pcDNA3.1-SARS-CoV-2-B.1.1.529-spike, pcDNA3.1-SARS-CoV-spike, pcDNA3.1-MERS-CoV-spike, pcDNA3.1-Bat-CoV-WIV1-spike, pcDNA3.1-Bat-CoV-Rs3367-spike, and pcDNA3.1-VSV-G) were all preserved in our laboratory.

EK1-C16 (SLDQINVTFLDLEYEMKKLEEAIKKLEESYIDLKEL-GSGSG-PEG4-C16) and EK1 (SLDQINVTFLDLEYEMKKLEEAIKKLEESYIDLKEL) were synthesized by Chengdu Shengnuo Biotechnology Co., Ltd. Authentic SARS-CoV-2 WT strain (nCoV-SH01, GenBank number: MT121215.1) was isolated and preserved in the Biosafety Level 3 (BSL-3) Facility of Fudan University. Authentic SARS-CoV-2 Omicron (hCoV-19/Hong Kong/HKU-344/2021) was isolated from a patient and maintained in the Biosafety Level 3 (BSL-3) Facility of the University of Hong Kong (HKU). HCoV-OC43 (VR-1558) was obtained from ATCC.

### 2.2. Authentic SARS-CoV-2 WT Strain Inhibition

Wild-type SARS-CoV-2 live virus inhibition assay was performed in the BSL-3 Facility, Fudan University. Briefly, peptides were first incubated with SARS-CoV-2 (100 TCID50) for 30 min and then added into the Vero-E6 cell line seeded in a 96-wall plate. After 1 h incubation, the supernatants containing peptide and SARS-CoV-2 were changed for fresh DMEM containing 5% FBS. After 48 h culture, Vero-E6 cells infected with SARS-CoV-2 were fixed with 4% paraformaldehyde, followed by 0.2% Triton X-100 treatment. Next, an immunofluorescence assay was performed to detect the nucleocapsid protein of SARS-CoV-2 in Vero-E6 cells [26]. The SARS-CoV-2 nucleocapsid antibody (1:200, Sino Biological, Beijing, China) was used as a primary antibody, the Alexa Fluor 488 goat anti-rabbit IgG (1:100, Thermo Fisher) was used as a secondary antibody, and DAPI (Thermo Fisher, Waltham, MA, USA) was used to stain the nucleus.

### 2.3. Authentic SARS-CoV-2 Omicron Variant Inhibition Assay

The inhibitory activity of peptides against SARS-CoV-2 isolate Omicron variant infection was assessed at HKU. Briefly, a diluted peptide was first incubated with 0.01 MOI Omicron variant (hCoV-19/Hong Kong/HKU-344/2021; GISAID accession number EPI_ISL_7357684) for 60 min. Next, this peptide–virus mixture was added into Vero-E6-TMPRSS2 cells which were seeded in a 96-well plate. After 72 h culture, CPE was observed and scored as 100% inhibition or 0% inhibition.

### 2.4. Package of Coronavirus Pseudovirus

Coronavirus PsVs were produced as previously reported. Briefly, HEK293T cells were seeded in a 6-well plate 24 h before transfection. Upon transfection, HIV backbone plasmid (pNL4-3.Luc.R-E) and spike-expressing plasmid, such as pcDNA3.1-SARS-CoV-2-spike, were co-transfected into HEK293T cells by Vigofect (Vigorous Biotechnology, Beijing, China). At 10 h post-transfection, cellular supernatants containing transfection reagent were changed for fresh DMEM containing 5% FBS. After another 36–48 h culture, cell supernatants containing PsV particles were collected and stored at −80 °C.

### 2.5. Coronavirus Pseudovirus Inhibition Assay

The inhibitory activity of peptides against pseudovirus infection was assessed as previously reported [18]. In brief, a serially diluted peptide was first incubated with pseudovirus for 30 min, and then this peptide–pseudovirus mixture was added into Caco2 cells seeded in a 96-well plate. After a 12 h culture, culture supernatants were discarded, and fresh DMEM was added. After another 36 h culture, luciferase assay (Promega, Madison, WI, USA) was performed to measure luciferase activity according to the manufacturer’s instructions. Inhibition curves were produced with GraphPad Prism 8 software, and IC_50_ values were calculated.

### 2.6. Authentic HCoV-OC43 Inhibition Assay

The inhibitory activity of peptides against authentic HCoV-OC43 infection was measured as previously reported [17]. A diluted peptide was first incubated with HCoV-OC43 (100 TCID50) for 30 min, and the peptide–virus mixture was added to the RD cell line seeded in a 96-well plate. The CCK-8 assay was used to assess cell viability by observing CPE of HCoV-OC43, and an inhibition curve was produced by GraphPad Prism 8 software.

### 2.7. Cell–Cell Fusion Inhibition Assay

A cell–cell fusion inhibition assay was performed as previously reported [17]. Briefly, HEK293T cells were transfected with plasmid pAAV-IRES-GFP-SARS-CoV-2-spike (or pAAV-IRES-GFP-MERS-CoV-spike, pAAV-IRES-GFP-HCoV-OC43-spike) to obtain 293T cells expressing GFP and SARS-CoV-2 spike protein (or MERS-CoV spike protein, HCoV-OC43-spike). A diluted peptide was then incubated with these transfected HEK293T cells for 30 min and added into target cells seeded in a 96-well plate. Two hours later, fusion status was observed using fluorescence microscopy.

### 2.8. Cytotoxicity Assay

The cytotoxicity of peptides was assessed as previously reported [27]. Briefly, a diluted peptide was co-incubated with RD cells seeded in a 96-well plate for 12 h. Next, the culture medium containing peptides was replaced with fresh DMEM. After another 36 h culture, the CCK-8 assay was used to assess cell viability.

### 2.9. Statistical Analysis

The inhibition curves and IC_50_ values of peptide inhibitors were all produced by GraphPad Prism 8 software.

## 3. Results

### 3.1. EK1-C16 Potently Inhibited Infection of SARS-CoV-2 Wild-Type (WT) Strain

**Figure 2:**
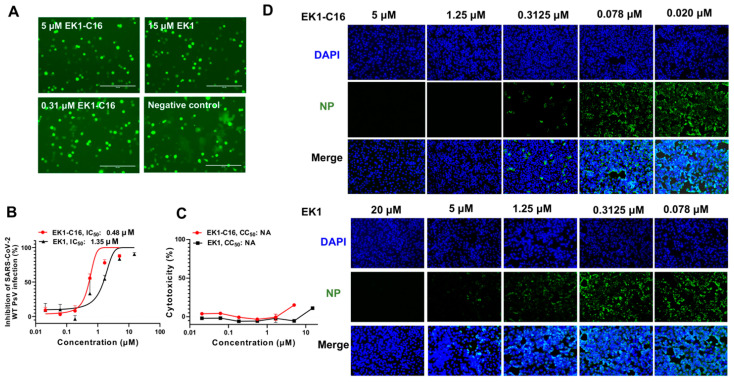
EK1-C16-mediated inhibition of SARS-CoV-2 infection. (**A**) EK1-C16-mediated inhibition of SARS-CoV-2 D614G S-meditated cell–cell fusion. (**B**) EK1-C16-mediated inhibition of SARS-CoV-2 WT (Wuhan-Hu-1) PsV infection. (**C**) Cytotoxicity of EK1-C16 to RD cells was tested by using a CCK-8 assay. (**D**) EK1-C16-mediated inhibition of authentic SARS-CoV-2 WT (nCoV-SH01) infection. Samples were tested in triplicate, and the experiment was repeated at least twice.

After designing and synthesizing the EK1-C16 lipopeptide, we first tested its inhibitory activity against SARS-CoV-2 D614G S-mediated cell–cell fusion and infection of the pseudotyped SARS-CoV-2 WT strain (Wuhan-Hu-1). We found that EK1-C16 at high (5.0 μM) and low (0.31 μM) concentrations could suppress SARS-CoV-2 S-mediated cell–cell fusion (Figure 2A). It also effectively inhibited SARS-CoV-2 WT pseudovirus (PsV) infection in Caco2 cells in a dose-dependent manner with an IC_50_ (half maximal inhibitory concentration) of 0.48 μM (Figure 2B). We then assessed the potential cytotoxicity of EK1-C16 using the CCK-8 assay. At the concentration of 5 μM, it exhibited no significant cytotoxicity (Figure 2C). Next, we used an authentic SARS-CoV-2 inhibition assay to determine the inhibitory activity of EK1-C16 against infection of authentic SARS-CoV-2 WT strain (nCoV-SH01, GenBank number: MT121215.1); an immunofluorescence assay was used to detect SARS-CoV-2 N protein expression. As shown in Figure 2D, EK1-C16 at 0.31 μM could effectively inhibit authentic SARS-CoV-2 WT infection. While EK1 at 0.31 μM showed no significant inhibitory activity, it did inhibit authentic SARS-CoV-2 infection at 5.0 μM, suggesting that EK1-C16 is more effective than EK1 in inhibiting authentic SARS-CoV-2 infection.

### 3.2. EK1-C16 Inhibited Infection of SARS-CoV-2 VOCs, Including Omicron

**Figure 3:**
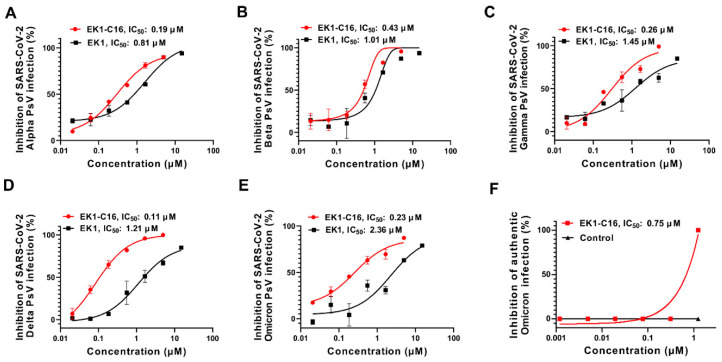
EK1-C16-mediated inhibition of infection by SARS-CoV-2 VOCs. EK1-C16-mediated inhibition of infection by pseudotyped SARS-CoV-2 Alpha (**A**), Beta (**B**), Gamma (**C**), Delta (**D**), and Omicron (**E**), and by authentic Omicron variant (**F**). The samples were tested in triplicate, and the experiment was repeated at least twice.

SARS-CoV-2 variants are constantly emerging. Some show increased infectivity and transmissibility, as well as reduced sensitivity to neutralization of therapeutic antibodies and vaccine-elicited sera. Here, we assessed the inhibitory activity of EK1-C16 against these SARS-CoV-2 VOCs. As shown in Figure 3A–E, EK1-C16 could effectively inhibit infection by pseudotyped SARS-CoV-2 VOC Alpha, Beta, Gamma, Delta, and Omicron with IC_50_ values of 0.19, 0.43, 0.26, 0.11, and 0.23 μM, respectively, which are about 3- to 10-fold more potent than that of SARS-CoV-2 WT. We further determined the inhibitory activity of EK1-C16 against infection of the authentic Omicron variant in Vero-E6-TMPRSS-2 cells by detecting the cytopathic effect (CPE) at 72 h post-infection. We found that EK1-C16 could effectively inhibit authentic Omicron infection with an IC_50_ value of 0.75 μM (Figure 3F).

### 3.3. EK1-C16 Broadly Inhibited Infection by Other Sarbecoviruses

**Figure 4:**
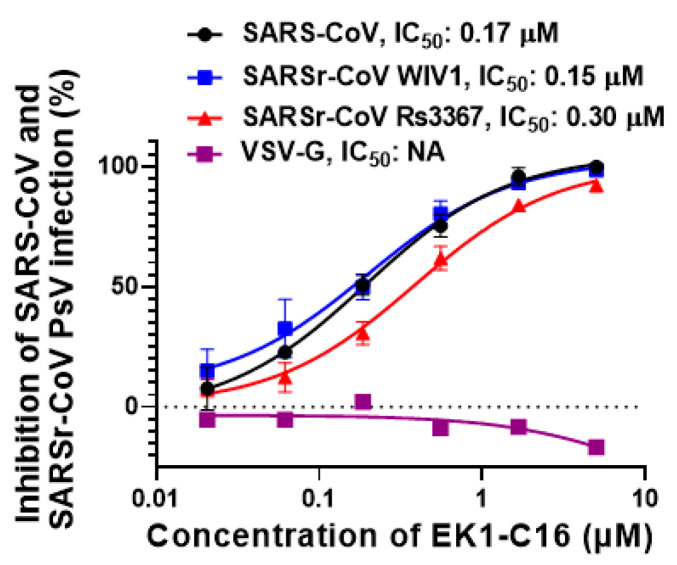
EK1-C16 can broadly inhibit sarbecoviruses. EK1-C16 can inhibit SARS-CoV, WIV1, and Rs3367 PsV, but it has no inhibitory activity against VSV-G PsV-mediated infection, indicating its specificity for sarbecoviruses. Each peptide inhibitor was tested in duplicate, and experiments were repeated twice.

SARS-CoV has the potential to re-emerge in the future, while bat SARSr-CoVs may cause emerging SARS-like infectious diseases in the future [28]. To prepare for these emerging or re-emerging coronavirus infectious diseases, it is essential to develop broad-spectrum antivirals. Here, we assessed the inhibitory activity of EK1-C16 against infection by pseudotyped SARS-CoV and bat SARSr-CoVs. We found that EK1-C16 could potently inhibit SARS-CoV PsV infection with an IC_50_ of 0.17 μM and bat SARSr-CoV WIV1 and Rs3367 infection with IC_50_ of 0.15 and 0.3 μM, respectively (Figure 4). In contrast, EK1-C16 exhibited no significant inhibitory activity against VSV-G PsV infection at a concentration as high as 5.0 μM (Figure 4), suggesting that the antiviral activity of EK1-C16 is specific for coronaviruses.

### 3.4. EK1-C16 Inhibited MERS-CoV Infection

**Figure 5:**
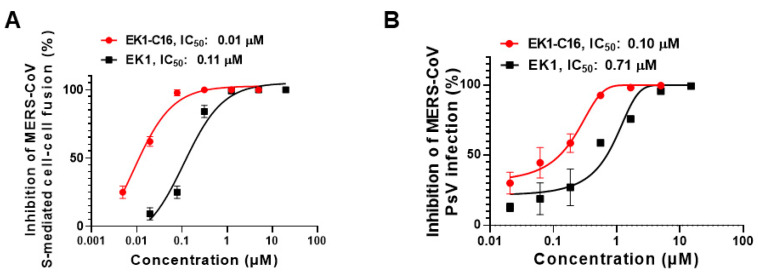
EK1-C16 can broadly inhibit MERS-CoV infection. (**A**) Inhibitory activity of EK1-C16 against MERS-CoV spike protein-mediated cell–cell fusion. (**B**) Inhibitory activity of EK1-C16 against MERS-CoV PsV. Samples were tested in triplicate, and the experiment was repeated twice.

Another highly pathogenic HCoV in human circulation is MERS-CoV. Although its infectivity and transmissibility are much lower compared to SARS-CoV-2, its case-fatality rate is as high as 34% [29]. Therefore, it is also essential to develop antivirals against MERS-CoV infection. Here, we first assessed the inhibitory activity of EK1-C16 against MERS-CoV S-mediated membrane fusion. We found that it could potently inhibit MERS-CoV S-mediated cell–cell fusion with an IC_50_ of 0.012 μM (Figure 5A), indicating nearly 10-fold more efficacy than that of EK1 peptide. Next, we evaluated the inhibitory activity of EK1-C16 against MERS-CoV PsV infection in Caco2 cells and found that it inhibited MERS-CoV PsV infection with an IC_50_ of 0.10 μM, about sixfold more potent than that of EK1 (Figure 5B). These results suggest that EK1-C16 could be further developed as a candidate antiviral for the prevention and treatment of MERS-CoV infection.

### 3.5. EK1-C16 Inhibited HCoV-OC43 Infection

**Figure 6:**
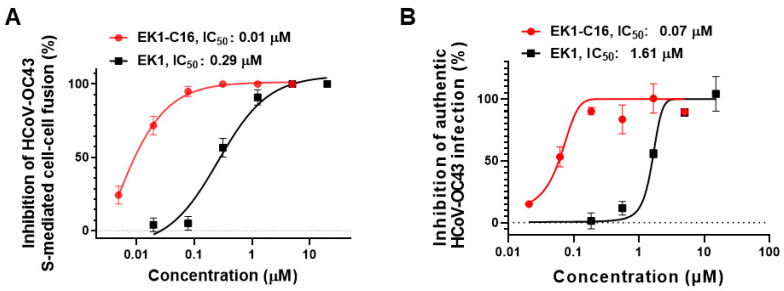
EK1-C16 can broadly inhibit HCoV-OC43 infections. (**A**) Inhibitory activity of EK1-C16 against HCoV-OC43 S-mediated cell–cell fusion. (**B**) Inhibitory activity of EK1-C16 against authentic HCoV-OC43 infection. The samples were tested in triplicate and the experiment was repeated once.

Apart from the above highly pathogenic HCoVs, some HCoVs with low pathogenicity, such as HCoV-OC43, continue to circulate widely in humans during the winter months and cause upper and respiratory tract illness and common cold-like symptoms [7,8]. HCoV-OC43 infection may also be associated with acute exacerbation of chronic obstructive pulmonary disease (AECOPD) and pneumonia in all age groups with immunocompromised conditions [8]. Therefore, it is also important to develop antivirals against HCoVs showing low pathogenicity [9,10]. Accordingly, in this study, we first measured the inhibitory activity of EK1-C16 against HCoV-OC43 S-mediated cell–cell fusion and found that EK1-C16 can potently inhibit HCoV-OC43 S-mediated cell–cell fusion with an IC50 value of 0.01 μM, which is about a 28-fold improvement compared to EK1 (Figure 6A). Next, we measured the inhibitory activity of EK1-C16 on the authentic HCoV-OC43 infection in RD cells. As shown in Figure 6B, EK1-C16 exhibited highly effective in inhibiting HCoV-OC43 infection with IC_50_ of 0.07 μM, about 22-fold more potent than that of EK1, indicating that EK1-C16, if well-developed, can also be used as a prophylactic or therapeutic against low pathogenic HCoV infection.

## 4. Discussion

The outbreak of COVID-19 sparked the development of a broad spectrum of antivirals, including therapeutic monoclonal antibodies, protein-, peptide- and small-molecule compound-based inhibitors, against SARS-CoV-2 infection [12,30,31]. However, the newly emerged SARS-CoV-2 VOCs, such as Omicron, have shown increasing resistance to some developed antiviral treatments and, even more concerning, SARS-CoV-2 RBD-specific neutralizing antibodies and vaccines being used worldwide [6,32,33,34,35]. The presence of SARSr-CoVs in bats may cause future outbreaks of SARS-like infectious diseases [28]. Thus, the growing list of SARS-CoV-2 VOCs and other emerging sarbecoviruses calls for the urgent development of antivirals with broad applicability and improved anti-coronavirus activity. It should be noted that MERS-CoV is still circulating in the Middle East region [36]. Several cases of SARS-CoV-2 and MERS-CoV co-infection were identified in Saudi Arabia [37], and both SARS-CoV-2 and MERS-CoV could infect type-II alveolar cells [38]. Furthermore, co-infection of immunocompromised individuals—for instance, by SARS-CoV-2 Omicron or MERS-CoV—could lead to a new species through genetic recombination [29]. Such an event could potentially increase the transmissibility of the current Omicron variant and reduce, even further, the sensitivity to SARS-CoV-2 neutralizing antibodies, while gaining a higher case-fatality rate (CF) of MERS-CoV. Such a scenario would spell disaster in countries with a low COVID-19 vaccination rate. Therefore, it is essential to develop highly effective pan-CoV therapeutics or prophylactics [10].

Our previous studies have shown that the HR1 domain is an important target for the development of potent and broad-spectrum HCoV fusion inhibitors [27,39]. We found that EK1 peptide targeting the HR1 domain of divergent HCoVs could broadly and effectively inhibit infection of all HCoVs and bat SARSr-CoVs tested [27]. Our cholesterol-conjugated EK1 lipopeptide, EK1C4, showed significant improvement in its inhibitory activity against SARS-CoV-2 and its VOCs, including Omicron [17,19]. However, while no cholesterol-based lipopeptides are currently in clinical use, some C16-based lipopeptide drugs are in clinical trials, showing the practicality of developing a C16-conjugated lipopeptide drug, as we have herein reported.

Specifically, our C16-conjugated, lipopeptide-based pan-CoV fusion inhibitor, EK1-C16, effectively inhibited infection by SARS-CoV-2 WT and its VOCs, including Omicron, with the highest transmissibility and lowest sensitivity to SARS-CoV-2 neutralizing antibodies. EK1-C16 lipopeptide is also highly effective against infection by SARS-CoV and bat SARSr-CoVs, MERS-CoV, and HCoV-OC43. Similarly, some small-molecule antivirals targeting the conserved region of other viral proteins of SARS-CoV-2, such as remdesivir and molnupiravir targeting viral RdRp and nirmatrelvir targeting Mpro, also exhibit broad-spectrum anti-HCoV activity and can potently inhibit infection from the Omicron variant [35,40]. Combinations of EK1-based peptides with these inhibitors targeting the conserved regions of other viral proteins are expected to have synergistic antiviral activity against infection of SARS-CoV-2 variants and other HCoVs.

Taken collectively, these results suggest that EK1-C16 is a highly promising candidate for development as a potent and broad-spectrum anti-HCoV drug for the prevention and treatment of infection by current and future SARS-CoV variants, as well as emerging and re-emerging coronaviruses.

## Data Availability

The raw data of this paper are available on request from the corresponding author.
